# Development and in vitro evaluation of a lignin-PLGA nanocarrier for florfenicol delivery

**DOI:** 10.1007/s11259-024-10595-7

**Published:** 2024-11-18

**Authors:** Emilia Trif, Carlos E. Astete, Sumit Libi, Emoke Pall, Septimiu Tripon, Cristina Coman, Diana Olah, Adrian Valentin Potârniche, Cristina M. Sabliov, Constantin Cerbu

**Affiliations:** 1 Department of Infectious Diseases, University of Agricultural Sciences and Veterinary Medicine, Cluj‑Napoca, Romania; 2 Department of Biological and Agricultural Engineering, Louisiana State University, Baton Rouge, LA 70803 USA; 3 Department of Molecular Biology and Biotechnology, Electron Microscopy Laboratory, Biology and Geology, Faculty, Babes-Bolyai University, Cluj‑Napoca, Romania; 4 Electron Microscopy Integrated Laboratory, National Institute for Research and Development of Isotopic and Molecular Technologies, Cluj‑Napoca, Romania; 5 Faculty of Food Science Technology, University of Agricultural Sciences and Veterinary Medicine, Cluj-Napoca, Romania

**Keywords:** Nanoparticles, Lignin, Antibiotic resistance, Florfenicol

## Abstract

Florfenicol (FF) is a widely used antimicrobial in veterinary medicine because of its broad antimicrobial activity, although it has certain limitations and raises concerns about the development of antimicrobial resistance genes. These limitations highlight the need to explore novel drug with controlled release systems to enhance the therapeutic efficacy of FF, while minimizing the potential for resistance development. This study introduces an innovative approach for the design, synthesis, and evaluation of lignin-poly(lactic-co-glycolic) acid (PLGA)-FF nanoparticles. By leveraging the properties of PLGA and lignin, this study aimed to augment the solubility, stability, and bioavailability of FF, thereby enabling dosage reduction and consequently diminishing the likelihood of resistance emergence and other limitations. Lignin-PLGA nanoparticles encapsulating FF were synthesized and characterized to assess their physicochemical properties, such as particle size, zeta potential, and drug loading efficiency. The release profile, antimicrobial efficacy, and cytotoxicity were evaluated. Comparative analyses with standard FF formulations were performed to ascertain the superior performance and potential benefits of the nanoparticle-based antimicrobials. Our findings indicate that the synthesized lignin-PLGA nanoparticles exhibited favorable drug delivery attributes, including a controlled and sustained release mechanism, significantly enhanced antimicrobial activity at reduced concentrations relative to free FF, with minimal cytotoxic effects. Importantly, the nanoparticle system inhibited bacterial biofilm formation, which is a key factor in the onset and spread of antimicrobial resistance. These findings underscore the potential of integrating biodegradable polymers with natural compounds to forge innovative pathways in drug delivery, addressing critical challenges in veterinary medicine.

## Introduction

Florfenicol (FF), a synthetic amphenicol-class antibiotic, has emerged as a critical antimicrobial in veterinary medicine because it is exclusively designed for animal health and is effective against a large variety of bacteria (Wei et al. 2016). Beyond its antimicrobial effect, FF has shown potential for mitigating inflammation (Shuang et al. 2011), adding another dimension to its utility.

Moreover, despite its relative novelty (Trif et al. 2023b), the application of FF in preventive and metaphylactic treatments and as a growth enhancer in aquaculture has precipitated the early onset of resistance genes (González-Martín et al. 2011; Reda et al. 2013), underscoring the importance of judicious FF usage in animals.

Despite their utility and versatility, challenges such as poor water solubility and difficult oral delivery have led to the exploration of nanostructures as drug carriers to improve FF’s pharmacokinetic and pharmacodynamic profiles of FF (Cerbu et al. 2021; Devadasu et al. 2013). Nanoparticles loaded with FF offer innovative solutions to these challenges, proving particularly effective in antimicrobial therapies. Among the various nanocarriers, polymeric nanoparticles—especially those based on poly(lactic-co-glycolic) acid (PLGA) have garnered significant interest due to their biocompatibility, biodegradability, and capability for controlled drug release (Dahier et al. 2012; Dupuis et al. 2022). Other systems, such as nanoemulsions (Zhang et al. 2016) and those utilizing natural polymers like chitosan (Qi et al. 2018) and albumin (Arriagada et al. 2019), also hold considerable promise in this context. Building on the foundation laid by prior research (Trif et al. 2023a), which highlighted the innovative use of nanotechnology to enhance the delivery and efficacy of FF in combating bacterial pathogens in veterinary applications, this study sought to explore new frontiers in antibiotic delivery. Trif et al. 2023a demonstrated the potential of nanodelivered FF to slow antimicrobial resistance (AMR) by reducing the required dosage of FF, while simultaneously enhancing its pharmacological properties. This achievement, marked by a decrease in the minimum inhibitory concentration (MIC) needed to combat bacterial infections without a cytotoxic effect, underlines the role of nanotechnology in optimizing antibiotic performance without compromising safety. Leveraging these insights, this investigation focused on a novel polymeric compound, based on a graft PLGA-lignin amphiphilic polymer, to develop a distinctive nanocarrier system for FF. PLGA is well known for its biocompatibility, biodegradability, and ability to facilitate controlled drug release, making it an ideal candidate for pharmaceutical applications (Danhier et al. 2012; Dupuis et al. 2022). Lignin, a natural polymer abundant in plant cell walls, offers additional advantages owing to its antioxidant properties and its potential to enhance polymer matrix stability (Astete et al. 2020; Uraki and Koda 2015; Vinardell and Mitjans 2017), thereby offering a synergistic effect when combined with PLGA. This study aimed to develop a core-shell nanoparticle system utilizing PLGA in the core and lignin on the surface(Astete et al. 2020) for the delivery of FF. The primary objective was to leverage the advantageous properties of both PLGA and lignin to improve drug loading, release dynamics, stability, and antimicrobial efficacy. and to determine whether lignin-PLGA nanoparticles can further minimize the FF dosage required for therapeutic effects, extend the release period, and provide additional benefits that could potentially broaden the spectrum of FF activity and its utility in veterinary medicine. This comparative study aimed to contribute to the evolving field of nanotechnology-enhanced drug delivery, specifically targeting the challenges posed by AMR in veterinary settings. By assessing the efficacy, safety, and practical benefits of a lignin-PLGA-based structures for FF, this study contributes to the field by exploring a novel type of FF loaded nanoparticles that may not only addresses the limitations of current antibiotic therapies, but also paves the way for more sustainable and effective antimicrobial strategies, such as the use of antibiotic-loaded nanoparticles in veterinary medicine.

## Materials and methods

### Synthesis

The lignin-PLGA grafting method followed a previously described procedure (Astete et al. 2020). After the synthesis of the lignin-PLGA graft copolymer, the next phase of our study involved the formation of lignin-PLGA-FF nanoparticles (NPs). To achieve this, 900 mg of lignin-PLGA polymer was combined with 90 mg of FF ((Fisher Scientific, Pittsburgh, PA), and the mixture was dissolved in 14 mL of ethyl acetate (Sigma Aldrich, St. Louis, MO, USA) at room temperature under continuous stirring. Subsequently, the organic phase was poured into 150 mL of nanopure water previously saturated with ethyl acetate and mixed for 10 min at room temperature. This mixture underwent high-pressure homogenization using a microfluidizer M-110P apparatus (Microfluidics Corp., Westwood, MA) at 30,000 psi with four passes, all conducted at 4 °C. Following homogenization, ethyl acetate was removed via evaporation using a Rotavapor R-300 (Buchi Corporation, Switzerland) at 33 °C under vacuum for 80 min. To enhance cryoprotection, 1 g of trehalose (Fisher Scientific, Pittsburgh, PA) was incorporated into the sample before freeze drying the final sample with a Freezone 2.5 L (Labconco Corp., Kansas City, MO) at -80 °C for 42 h. The dry powder of stable lignin-PLGA-FF nanoparticles (lignin-PLGA-FF NPs) with preserved physicochemical properties was stored at -20 °C for subsequent characterization and evaluation.

### Characterization of the FF loaded NPs

The lignin-PLGA-FF NPs were characterized in terms of size, zeta potential, and size distribution, through dynamic light scattering, a method previously described (Trif et al. 2023a).

The morphology of the synthesized nanoparticles was examined using a previously described transmission electron microscopy method (Trif et al. 2023a).

### Release profile and kinetics

To evaluate the release kinetics of lignin-PLGA-FF NPs, a dialysis-based method was employed (Kang et al. 2017) Initially, 150 mg of lignin-PLGA-FF NPs were suspended in 15 mL of 10% phosphate-buffered saline (PBS) solution at pH 7.4. Subsequently, the suspension was distributed into three dialysis bags with a molecular weight cutoff of 12–14 kDa and flat width of 45 mm (Spectra/Por). At predetermined time intervals, 250 µl samples were withdrawn from each dialysis bag over 72 h (T0, T1,…, T12).

The extracts were filtered through a 0.45 μm Chromafil Xtra nylon filter, after which 20 µL of the filtrate was injected into a High-Performance Liquid Chromatography (HPLC) system. An Agilent 1200 HPLC system, comprising a quaternary pump, solvent degasser, autosampler, and UV-Vis detector with a photodiode (DAD), was usedfor the HPLC-DAD method. This method has been validated for similar applications (Teixeira et al. 2008; Patyra et Kwiatek 2019; Sandor et al. 2023).

Compounds were separated on a Kinetex XB C18 column (4.6 × 150 mm, 5 μm particles, Phenomenex, USA). Concerning the chromatographic conditions, the mobile phase consisted of methanol/water (50/50, v/v) with 0.1% formic acid, maintained for 10 min at 25 °C, at a flow rate of 1.0 mL/min, and chromatograms were captured at a wavelength of 260 nm. HPLC-grade methanol was procured from Supelco (Germany), and ultrapure water was obtained from a Direct-Q UV system (Millipore, USA). Formic acid (purity 99%) was obtained from VWR Chemicals (France).

A calibration curve was established by injecting seven different concentrations of FF (10, 20, 50, 200, 300, 400, and 500 µg/ml) dissolved in the mobile phase, which was repeated twice. The resulting curve equation, y = 2.255x– 6.4801, was used to quantify the concentration of FF in the analyzed samples. For the release study, the aforementioned dialysis procedure was repeated three times at different temperatures: 3 °C, 6 °C, and 21 °C. Each dialysis experiment was conducted in triplicate, independently, using fresh dialysis bags and PBS solutions at the respective temperatures. After the designated time points, the samples were withdrawn and processed following the same protocol described above for FF quantification via HPLC analysis.

The drug release profiles were subsequently evaluated using various kinetic models, including zeroth-order, first-order, Higuchi, and Korsmeyer-Peppas models. The suitability of each model was assessed based on regression coefficients to identify the model that best described the release behavior.

To quantify the amount of FF encapsulated within the nanostructures, the initial values (T0) obtained from the release assay were used. Entrapment efficiency (EE) and loading capacity (LC) were calculated using the following equations:

$$\begin{aligned} & Loading\;capacity\left(LC\right)= \\& \frac {amount\;at\;\frac{T0}{1000}\ast\;total\;weight\;of\;nanoparticles}{mass\;of\;nanoparticles\;at\;T0}\end{aligned}$$

$$\begin{aligned} & Entrapment\;Efficiency\;\left(EE\%\right) \\& =\frac{amount\;of\;total\;entrapped\;drug}{theoretical\;mass\;of\;drug}\end{aligned}$$

### Cell isolation and cytotoxicity assay

To determine the cytotoxic effect, primary cells from different animal species and a normal rainbow trout cell line were employed, either isolated from animal tissue samples (porcine primary fibroblast isolation, horse mesenchymal stem cells) or commercial cell lines (rainbow trout cell line, American Type Culture Collection, Manassas, VA, USA).

Primary porcine fibroblasts and peripheral blood mononuclear cells from horses were isolated using a previously described method (Trif et al. 2023a).

To test the cytotoxic effect on aquatic animals (fish), a rainbow trout cell line exhibiting epithelial-like morphology (RTgill-W1 cells, ATCC, CRL-2523, Manassas, VA, USA) was cultured according to the manufacturer’s instructions (previously stored in liquid nitrogen, to ensure the highest survival rate of the cells), as described below. The vial was warmed by gently shaking it in a water bath set between 18 °C and 20 °C (avoiding the introduction of the O-ring and cap in the water to minimize the contamination risk) for approximately 2 min. Once the contents were fully thawed, the vial was promptly removed from the water bath and sterilized by dipping in 70% ethanol. Next, the contents were transferred into a centrifuge tube that contained 9.0 mL of Leibovitz’s L-15 Medium (30–200, ATCC, Manassas, VA, USA) and centrifuged at approximately 125× g for 5 to 10 min. After centrifugation, the cell pellet was gently resuspended in Leibovitz’s L-15 medium and supplemented with 10% fetal bovine serum (Sigma Aldrich), then transferred into a culture flask, and incubated at a temperature range of 18 °C to 20 °C, without the addition of CO_2_.

The collection and use of the animal cell lines was approved by the UASVM CN ethics committee decision number 246/06.04.2021.

Cell viability in the presence of lignin-PLGA-FF NPs was evaluated using an MTT assay (Trif et al. 2023a; van Meerloo et al. 2011).

### Antimicrobial efficacy

The antibacterial efficacy of lignin-PLGA-FF NPs was assessed in comparison with free FF, with each sample tested in triplicate, to identify which formulations would demonstrate the most promising antimicrobial efficacy. This evaluation aimed not only to determine the MIC of these formulations, but also to investigate their capacity to prevent bacterial biofilm formation and disrupt pre-existing biofilms, utilizing both qualitative and quantitative testing methods.

A previously described method was used to determine the minimum inhibitory concentration (MIC) (Trif et al. 2023a, b).

Controlled bacterial strains were subjected to rigorous testing to ensure biofilm formation using a previously employed method (Trif et al. 2023a), modified after (Costa et al. 2018) thus, quantitative determination was performed using a modified crystal violet assay method, in order to evaluate the antibiofilm activity of the FF loaded nanoparticles.

### Scanning electron microscopy

To assess the efficacy of inhibiting bacterial biofilms using scanning electron microscopy (SEM), the experiment was replicated using the chosen ATCC bacterial strains along with free FF and lignin-PLGA-FF NPs, following the detailed method described in Sect. 4.2. To visualize the ultrastructure of the preformed biofilms, a previously employed method was used (Trif et al. 2023a).

### Statistical analyses

Statistical analyses and graphs were created using GraphPad Prism v.9.3.0 (GraphPad Software, San Diego, USA).

## Results

### Characterization

The synthetized nanoparticles had a mean diameter of 85.67±0.59 nm for empty lignin-PLGA NPs and 87.16±0.75 nm for lignin-PLGA-FF NPs. The polydispersity index (PDI) and zeta potential were 0.104±0.011 and − 55.97±3.43 mV for lignin-PLGA-FF NPs, and 0.089±0.020 and − 55.33±2.32 mV for empty lignin-PLGA NPs, indicating that particles were < 100 nm, monodisperse, and negatively charged.

**Fig. 1:**
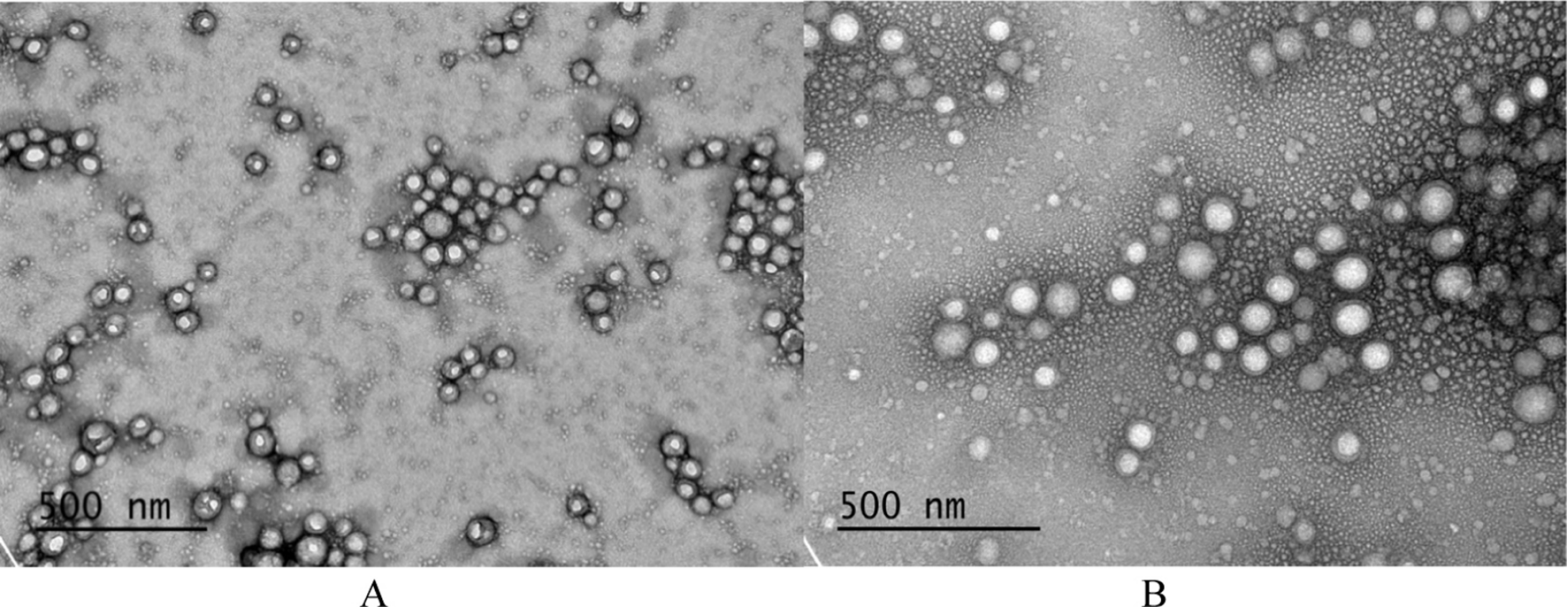
Transmission Electron Microscopy (TEM) images for empty lignin-PLGA NPs (**A**) and lignin-PLGA NPs (**B**)

Both empty nanostructures (lignin-PLGA and lignin-PLGA-FF NPs) were spherical with a narrow size distribution (as illustrated in Fig. 1).

**Fig. 2:**
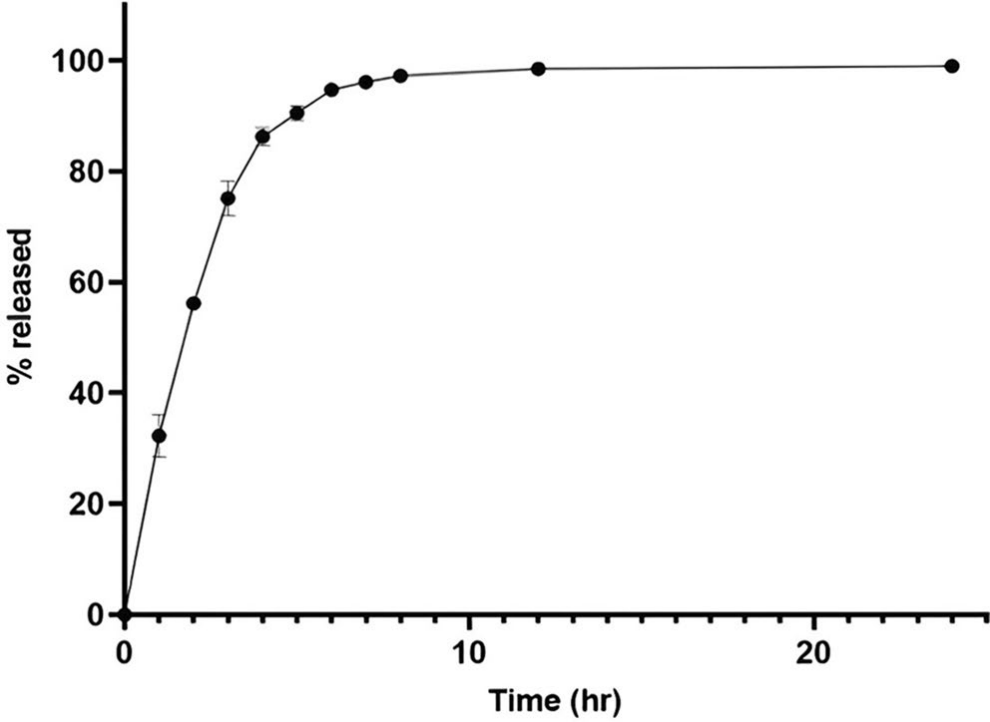
Release study of FF from lignin-PLGA-FF NPs

**Fig. 3:**
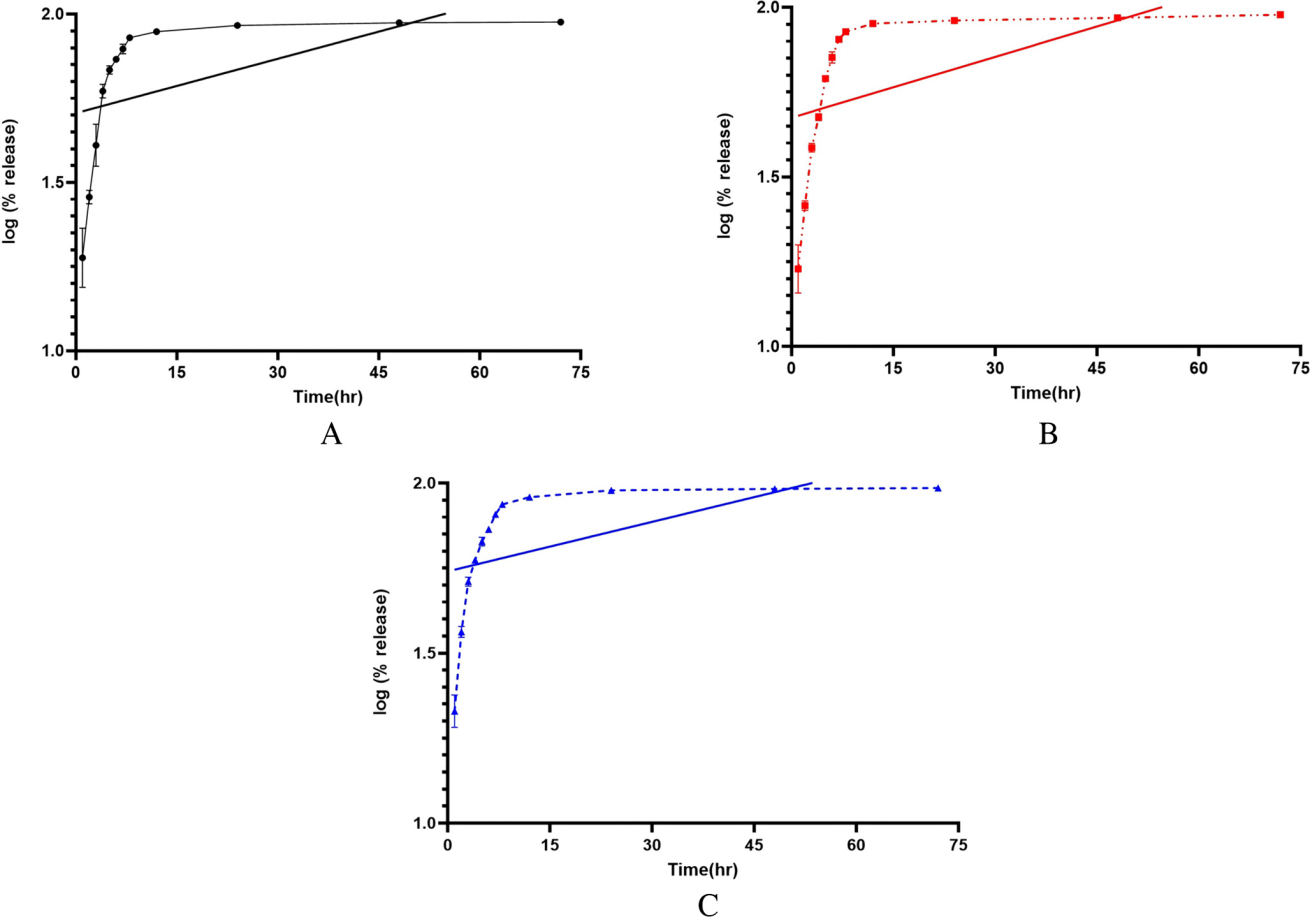
First order release kinetics of FF from lignin-PLGA-FF NPs at 3 °C (**A**), 6 °C (**B**) and 21 °C (**C**)

The release study of FF from lignin-PLGA-FF NPs (Fig. 2) indicated a sustained release of FF over 24 h, with relatively uniform drug diffusion. Drug kinetics were evaluated using various kinetic models, such as zeroth-order, first-order release model, Higuchi model, and Korsmeyer-Peppas models, at three different temperatures (3 °C, 6 °C, and 21 °C); and the regression coefficients are listed in Table 1. The first-order release model (Fig. 3) was identified as the most suitable option for the given formulations, as it most accurately characterized the drug release profile.

**Table 1:** Values of release constant (k) and regression coefficients (R^2^) for drug release profile with different mathematical models at different temperatures

	3 °C		6 °C		21 °C	
	k	*R* ^2^	k	*R* ^2^	k	*R* ^2^
Zeroth order	0.8812	0.3605	0.928	0.3824	0.868	0.3652
First order	0.005382	0.2667	0.005975	0.285	0.004851	0.2822
Hixon-Crowell	0.03898	0.5341	0.04162	0.544	0.05107	0.57
Higuchi	10.35	0.61	10.75	0.629	10.23	0.6219
Korsmeyer-Peppas	0.3524	0.7027	0.3852	0.727	0.3144	0.7283

The loading capacity for the lignin-PLGA-FF NPs was 53.26 µg/mg, with an entrapment efficiency of 60.38%.

### Antimicrobial activity- minimum inhibitory concentration (MIC)

Following the exposure of the three distinct bacterial strains, *Staphylococcus aureus* (ATCC 29213), *Escherichia coli* (ATCC 25922), and *Pseudomonas aeruginosa* (ATCC 27853), to free FF and lignin-PLGA-FF NPs, a comparative analysis was conducted on the antibacterial efficacy of these treatments by assessing their MIC. The evaluation revealed that lignin-PLGA-FF NPs achieved a significant reduction in the MIC values: approximately 99.84% for *S. aureus*, 99.84% for *E. coli*, and 99.96% for *P. aeruginosa*, relative to the MIC values observed with the free form of FF (Table 2). This significant decrease underscores the enhanced antibacterial potency of lignin-PLGA loaded with the FF nanoparticles.

**Table 2:** Minimum inhibitory concentration (MIC) of FF and lignin-PLGA-FF NPs for the three selected ATCC bacterial strains

Bacterial strains	FF (µg/mL)	FF from lignin-PLGA-FF NPs (µg/mL)
*S. aureus* (ATCC 25923)	0.244 ± 0.018	0.002 ± 0.0004
	Reduction of **99.84%** (mean value presented, *n* = 3)	
*E.coli* (ATCC 25922)	0.122 ± 0.03	0.002 ± 0.0002
	Reduction of **99.84%** (mean value presented, *n* = 3)	
*P. aeruginosa* (ATCC 27853)	0.488 ± 0.0021	0.019 ± 0.0002
	Reduction of **99.96%** (mean value presented, *n* = 3)	

### Antibiofilm activity

#### Inhibition of initial biofilm formation

The comparative effectiveness of free FF and FF delivered by lignin-PLGA-FF nanoparticles in inhibiting biofilm formation by the selected bacterial strains indicated that the encapsulation of FF within lignin-PLGA-FF nanoparticles influences the biofilm inhibition capacity, with varying degrees of effectiveness observed across different bacterial strains (Table 3). The negative percentages of inhibition suggest that the read value is higher than the control value, which could indicate an increase in biofilm formation.

**Table 3:** Percentage of the antibiofilm activity identified when testing FF and active FF from lignin-PLGA-FF NPs (mean values presented, *n* = 3)

Bacterial strains			FF		FF from lignin-PLGA-FF NPs	
*S. aureus* (ATCC 25923)			78.02%		70.79%	
*E.coli* (ATCC 25922)			90.05%		70.89%	
*P. aeruginosa* (ATCC 27853)			61.10%		52.29%	
Concentration of FF tested/ FF from lignin-PLGA-FF NPs (µg/mL)	*Staphylococcus aureus*		*Escherichia coli*		*Pseudomonas aeruginosa*	
	FF	FF from lignin- PLGA-FF NPs	FF	FF from lignin- PLGA-FF NPs	FF	FF from lignin- PLGA-FF NPs
125/ 6.65	**78.02%**	**70.79%**	**90.05%**	**70.89%**	**61.10%**	**52.29%**
62.5/3.32	50.09%	70.01%	88.59%	69.6%	23.96%	49.0%
31.25/1.66	-12/9%	67.9%	20.03%	63.0%	14.17%	23.7%
15.62/0.83	-25.29%	15.3%	-1.61%	65.8%	24.38%	22.0%
7.81/0.41	-41.6%	9.97%	16.97%	61.5%	22.29%	13.4%
3.90/0.20	-25.4%	14.2%	-37.14%	59.3%	21.88%	7.6%
1.95/0.10	-23.8%	12.0%	-39.80%	43.9%	27.71%	13.4%
0.97/0.05	-14.2%	7.05%	-21.21%	43.9%	16.46%	-13.6%
0.48/0.02	-9.7%	13.4%	-18.99	28.2%	17.5%	-17.8%
0.24/0.01	0.7%	19.5%	-4.44%	20.6%	8.33%	-21.7%
0.12/0.006	-7.6%	15.9%	-35.35%	19.6%	2.71%	-17.19%
0.06/0.003	-37.2%	16.7%	-19.80%	13.2%	-8.96%	-21.7%
0.03/0.0016	-12.3%	2.99%	-26.06%	18.4%	-2.29%	-18.8%
0.015/0.0008	-18.89%	3.98%	5.94%	27.3%	4.39%	-22.5%
0.007/0.0004	1.19%	4.76%	9.80%	14.7%	6.04%	-6.5%
0.003/0.0002	-16.3%	-2.719	16.06%	12.9%	-16.25	-12.5%

Compared with a solution of PLGA-FF NPs (Trif et al. 2023a), the results revealed a higher inhibition of the tested biofilm when testing the same concentration of thenanoparticle solution. Although the starting solution concentration was set at 125 µg/mL for all three formulations, the actual amount of FF available for antimicrobial activity varied significantly. Specifically, the PLGA-FF NPs contain 0.28 µg/mL of FF, whereas the lignin-PLGA-FF NPs had a higher FF content of 6.65 µg/mL. The free FF formulation implicitly has the entire concentration of active FF (125 µg/mL) because there is no encapsulation involved.

**Fig. 4:**
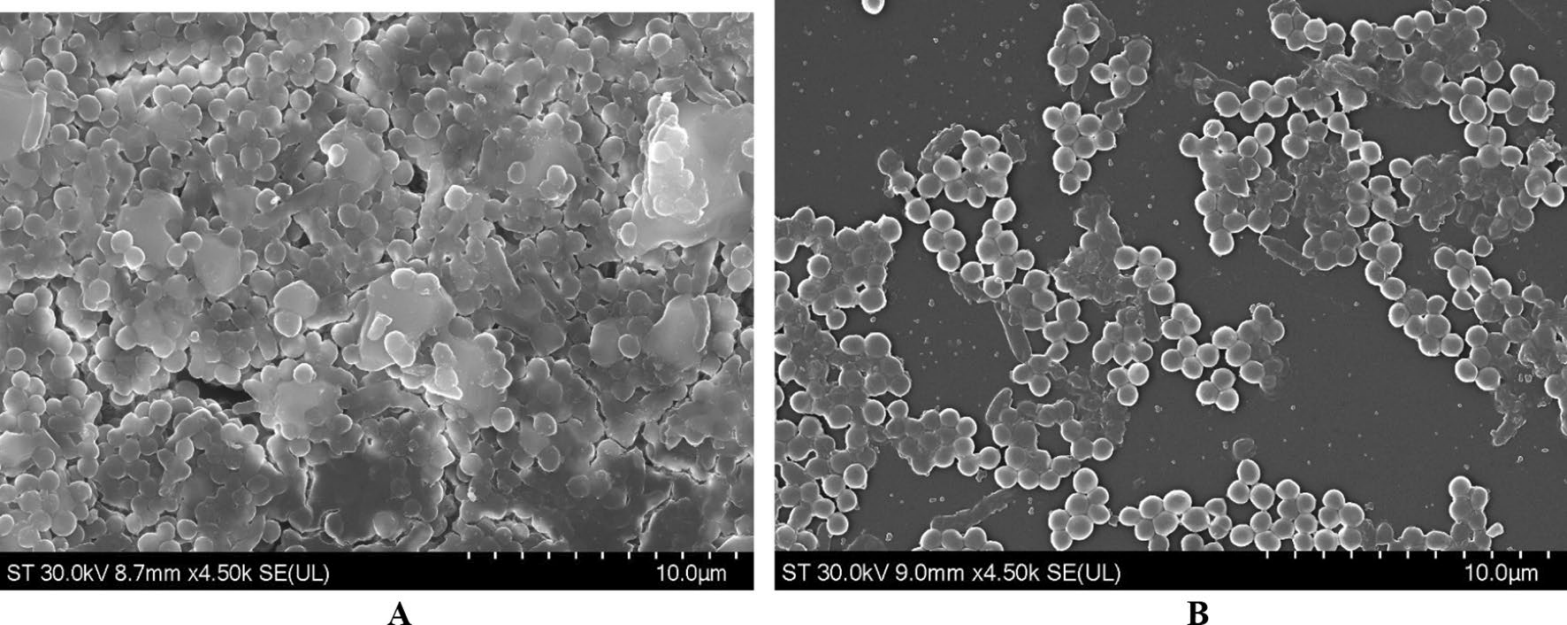
SEM images of *S.aureus* biofilm (**A**) and *S.aureus* biofilm when treated with a solution of 125 µg/mL lignin-PLGA-FF NPs (**B**) (10 µm)

**Fig. 5:**
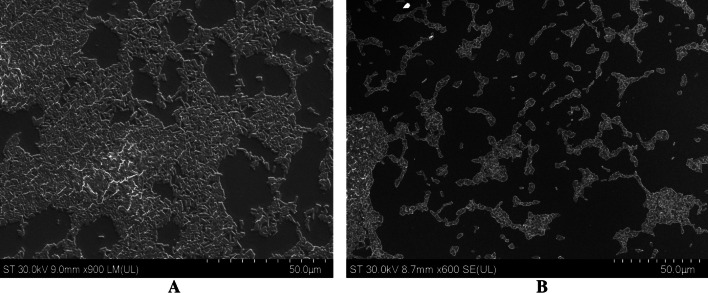
SEM images of *E.coli* biofilm (**A**) and *E.coli* biofilm when treated with a solution of 125 µg/mL lignin-PLGA-FF NPs (**B**) (50 µm)

**Fig. 6:**
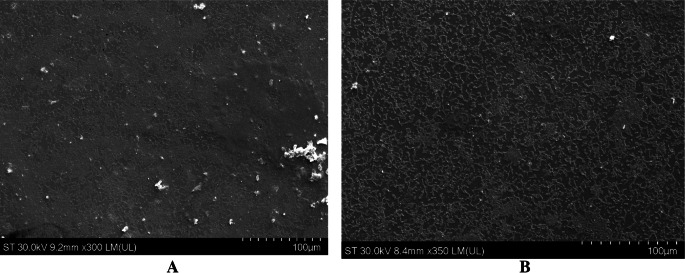
SEM images of *P. aeruginosa *biofilm (**A**) and *P.aeruginosa *biofilm when treated with a solution of 125 µg/mL lignin-PLGA-FF NPs (**B**) (100 µm)

The SEM images (Figs. 4, 5 and 6) provide compelling visual evidence that supports the data previously provided that the nanoparticle formulation containing FF (lignin-PLGA-FF NPs) has an inhibitory effect on biofilm formation across the three tested bacterial strains. Compared to the control samples, where biofilm formation by each of the cultivated bacteria was observed in its native and unimpeded state, the samples treated with lignin-PLGA-FFNPs exhibited disruption in biofilm integrity and density. This disruption reinforces the potential of the nanoparticle system as a promising strategy for the fight against biofilm-associated infections.

#### Antibiofilm properties when treating a preformed biofilm

Although the impact on the preformed biofilms was assessed by introducing both the antibiotic in its free form (solution of FF) and encapsulated within the nanoparticle solution (lignin-PLGA-FF NPs), the intervention did not lead to complete eradication of the biofilms, as the spectrophotometric readings were comparable to those of the positive control (biofilm formed by the tested bacteria in its native way). Statistical analysis indicated no significant difference (*p* > 0.05) between the efficacies of the tested concentrations.

### Cell citotoxicity assasy-MTT

**Fig. 7:**
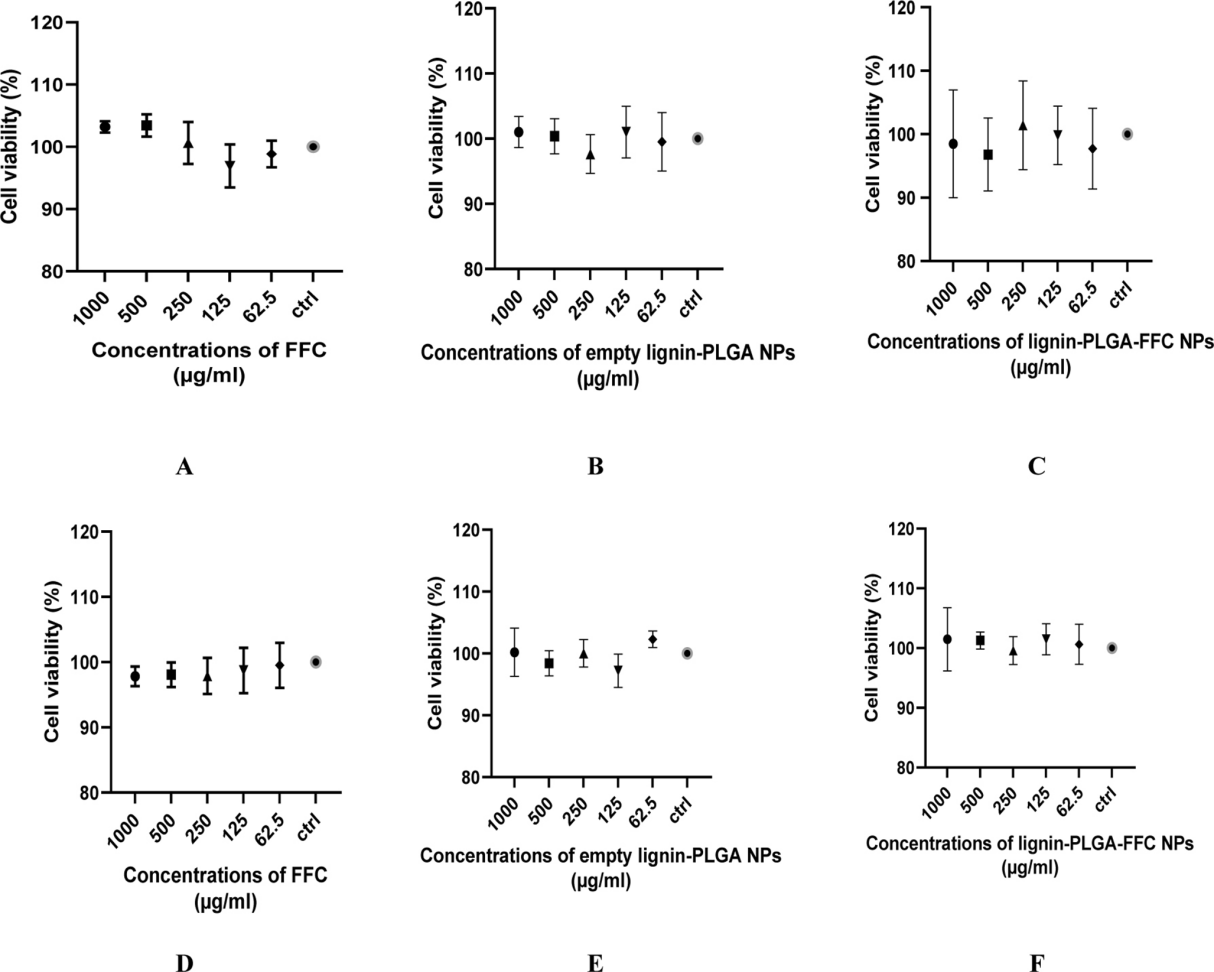
**A**, **B**, **C** MTT assay on porcine primary fibroblasts at five different concentrations of FF, lignin-PLGA and lignin-PLGA-FF NPs; **D**, **E**, **F** MTT assay on horse mesenchymal stem cells at five different concentrations of FF, lignin-PLGA and lignin-PLGA-FF NPs. (mean values presented, *n* = 3)

The MTT assay of porcine fibroblasts and equine mesenchymal stem cells treated with five different concentrations of FF, empty lignin-PLGA NPs, and lignin-PLGA-FF NPs indicated no morphological modifications, no loss in cell viability, and no cytotoxic potential. In addition, they did not present a dose-dependent effect, based on the statistically insignificant difference (p value from the 2 way ANOVA test was 0.8862 and 0.5793 from the ANOVA test) between the results obtained at different concentrations. All data are presented as the mean value ± standard error of the mean, as shown in Fig. 7.

**Fig. 8:**
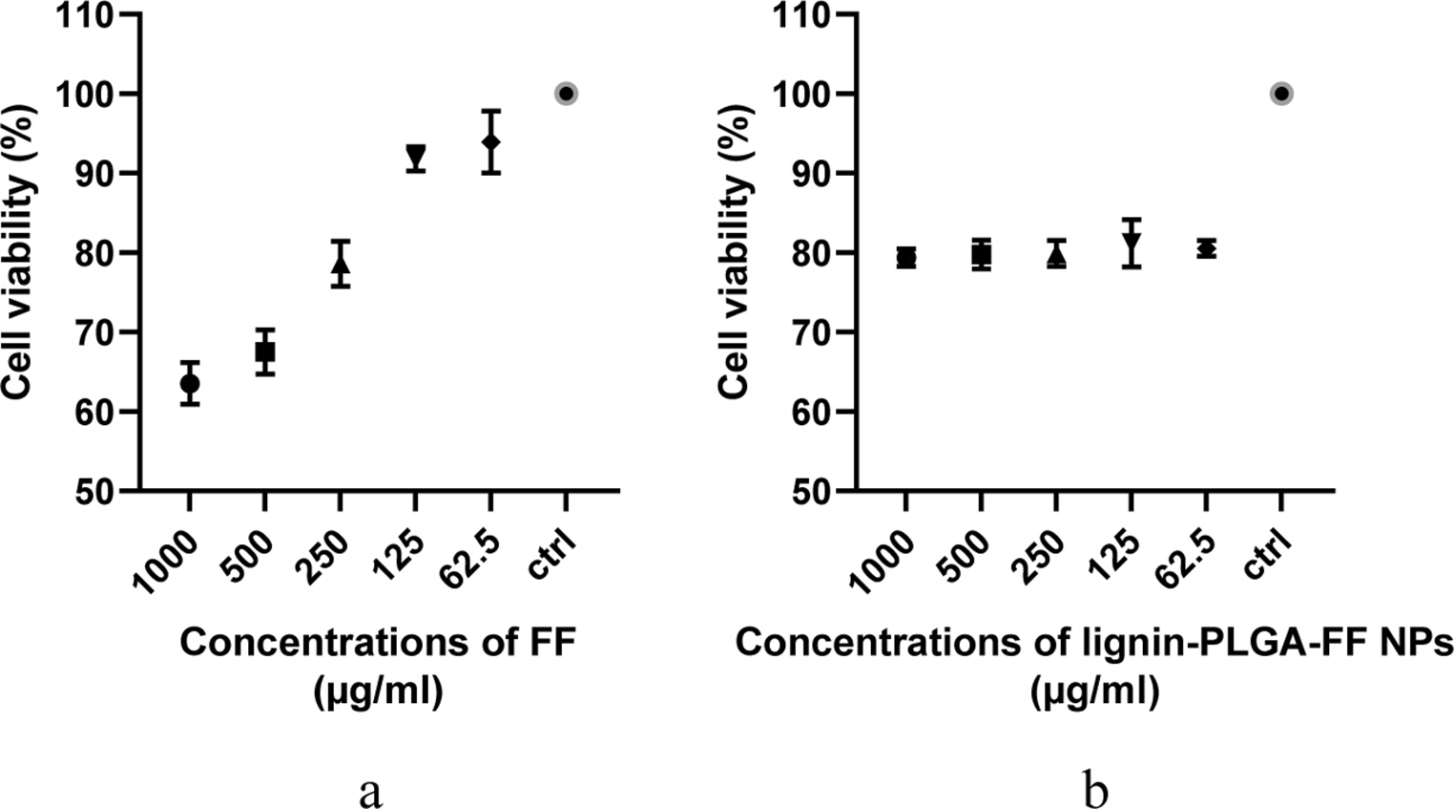
MTT assay on fish cells and six different concentrations of FF (**a**) and PLGA-lignin-FF NPs (**b**); (mean values presented, *n* = 3)

When tested on fish cells (rainbow trout cells), the lignin-PLGA-FF NPs decreased cell proliferation compared to the positive control, as graphically illustrated in Fig. 8. Additionally, morphological alterations, including cluster formation, decreased viability, and the appearance of deposits resembling sand, were observed. However, statistically significant differences were not observed (*p* = 0.1057 from the ANOVA test) between the different concentrations tested, suggesting no dose-related differences in cell proliferation. Free FF was also observed to induce a reduction in cell proliferation, consistent with the existing literature that highlights its cytotoxic effects on fish, even at therapeutic dosages (Bardhan et al. 2023; Mallik et al. 2023). The obtained data for free FF illustrated a dose-dependent effect, as there was a significant difference (*p* = 0.001 from the ANOVA test) in cell proliferation across various concentrations (from 62.5 to 1000 µg/mL), as illustrated in Fig. 8, reinforcing the importance of minimizing the FF dosage in aquaculture.

## Discussion

### Size, size distribution, and zeta potential

The mean diameters of the empty lignin-PLGA NPs (85.67±0.59) and lignin-PLGA-FF NPs (87.16±0.75) highlight the small nanoparticle sizes. The drug was encapsulated within the nanoparticles (loading capacity of 53.26 µg/mg) without altering their size, which is an important aspect of cellular uptake (Albanese et al. 2012). Considering that nanoparticles in the range of 1–100 nm are generally associated with improved cellular uptake compared to larger nanoparticles (Desai et al. 1997), but also smaller sizes (NPs smaller than 10 nm), which might show rapid clearance through renal filtration (Hoshyar et al. 2016), the obtained size is within the range that could be beneficial for an antibiotic delivery system. Furthermore, the size of the obtained nanoparticles was in the range of those that could undergo endocytosis (Sousa De Almeida et al. 2021). In addition, the size and shape of the NPs are critical in determining their internalization efficiency and drug release profile (Adamo et al. 2017). The obtained shape (spherical) is favorable, as spherical NPs are generally considered to have more efficient uptake than rod-shaped NPs (Champion and Mitragotri 2006; Chithrani et al. 2006).

The polydispersity index (PDI) is a critical indicator of the range and diversity within the particle size distribution (Raval et al. 2018). The obtained value (0.104 ± 0.011) indicated that the particles were monodisperse (PDI < 0.2). The zeta potential, the term used for the electrokinetic potential in colloidal systems and also a measure of surface charge (Gumustas et al. 2017), falls well within the stable range (more than + 30 mV and less than − 30 mV) (Honary and Zahir 2013), suggesting that the nanostructures are highly stable. The high zeta potential obtained is indicative of a strong repulsive force that dominates the attractive force, thereby preventing particle aggregation (Beck-Broichsitter et al. 2011; Harush-Frenkel et al. 2010). The obtained value for zeta potential (-55.97 ± 3.43 mV) also suggests the ease of redispersion, which is another critical aspect of nanocarrier formulations (Alshawwa et al. 2022; Shrestha et al. 2020). This is beneficial for the long-term storage and handling of nanocarrier formulations, where sedimentation might occur over time, and also ensures that, upon agitation, the particles will easily redisperse, maintaining the formulation’s effectiveness.

The lignin-PLGA-FF NPs synthesized in this study demonstrated a smaller mean diameter of 86.30 nm, in contrast to the larger dimensions reported in previous studies for 101 nm (Trif et al. 2023a), and 115.32 nm to 130.83 nm (Karp et al. 2019) for PLGA-FF NPs. It is important to note that lignin-PLGA-FF NPs were synthesized from an amphiphilic polymer without the aid of surfactants, whereas PLGA NPs reported in the literature were prepared in the presence of surfactants. The reduced size of the lignin-PLGA-FF NPs, which may enhance tissue penetration and increase the surface area-to-volume ratio, suggests potential advantages for drug release and underscores the suitability of the NPs for antibacterial delivery systems.

Overall, the obtained nanostructures possess characteristics that are favorable for biomedical applications, with the potential to contribute to improved therapeutic outcomes in veterinary medicine. The size, shape, stability, and drug encapsulation efficiency of these NPs suggest they could serve as effective platforms for the development of targeted treatment strategies.

### Release

A comparison of the release profiles of FF from different polymeric nanoparticle formulations revealed critical insights into the design and efficacy of these nanostructures for veterinary applications. Delayed release can interfere with the bioavailability of the antibiotic and can reduce the frequency of administration, a major aspect with clinical and economic implications in veterinary medicine (Trif et al. 2023a). The 24 h release profile of FF from lignin-PLGA-FF NPs may offer an advantage over the short half-life of free FF (2 h to 18 h under normal conditions), particularly in controlled conditions (Papich 2016; Park et al. 2008; Varma et al. 1986). In contrast, in a previously published study (Trif et al. 2023a), the PLGA formulation achieved a delayed release of FF for 6 h, with complete release occurring relatively quickly thereafter. While a delay in release was observed, further studies could explore whether optimizing polymer selection and FF loading strategies might extend this duration. The difference in release durations observed in this study and compared to previous data may indicate that the synthesis method plays a role, particularly through the lignin-PLGA matrix and deliberate omission of surfactants in this formulation. The use of lignin, a natural polymer that was (Uraki and Koda 2015) chemically modified with PLGA, could also contribute to the more extended and sustained release pattern of FF. The structural and chemical properties of lignin might influence the diffusion rate of antibiotics from the matrix, potentially contributing to a slower release rate (Miyazawa et al. 2021). This approach simplifies the formulation and may enhance the effectiveness of the nanodelivery system, although more research is needed to fully validate these findings.

### Cytotoxicity

Cytotoxicity assessment is a very important aspect when evaluating the nanoparticles as it signals potential toxic effects (Vinken and Blaauboer 2017). FF has been associated with cellular damage at the mitochondrial level, leading to cell death (Hu et al. 2017), thus highlighting the importance of comparing the cytotoxic effects of free FF with those of FF delivered via lignin-PLGA nanoparticles. For PLGA, the data are already known regarding the safety and biocompatibility (FDA approved for medical applications) (Makadia and Siegel 2011), and testing was considered necessary both for the evaluation of the lignin-PLGA polymer toxicity and the entire structure lignin-PLGA-FF NPs. Lignin-PLGA-FF NPs did not exhibit cytotoxic effects on isolated cells (horse mesenchymal stem cells, porcine primary fibroblasts, and rainbow trout epithelial cells) and did not show a dose-dependent effect. These findings suggest that the FF loaded NPs may be a viable candidate for in vivo testing to further investigate their safety and effectiveness. When tested on fish cell lines, while both free FF and nanodelivered FF reduced cell proliferation, the mechanisms and dose dependence appeared to differ. FF showed clear dose-dependent cytotoxicity, whereas the NPs did not show a statistically significant dose-response. This difference might be influenced by various factors, including the mode of delivery or cellular uptake. It is noteworthy that the mean cell proliferation percentage for lignin-PLGA-FF NPs (80.15%) was higher than the cell proliferation percentages presented by the first three tested concentrations for free FF, 63.51%, 66.2%, and 78.58%. This observation may indicate a reduction in antimicrobial toxicity when released from the nanoparticles. Further research is required to better understand the dynamics of the interaction with fish cells, including whether there is a threshold effect, the underlying mechanisms of cytotoxicity, and whether these effects are specific to a certain cell type or more broadly applicable. Additionally, the morphological changes observed warrant a deeper investigation into the pathways leading to these outcomes, potentially involving studies of apoptosis, oxidative stress, and other cellular responses to both free FF and NPs.

### Antimicrobial activity

Across all bacterial strains tested, the lignin-PLGA-FF NPs formulation consistently demonstrated the highest antimicrobial activity, indicated by its effectiveness at the lowest active FF concentrations.When compared to other nanodelivery systems, such as PLGA-FF NPs (Trif et al. 2023a), it suggests that the addition of lignin to the PLGA-FF NPs may enhance the antimicrobial potency of the nanoparticles. Although free FF showed good efficacy, the nanoparticle formulation outperformed the free drug in terms of the required concentration for bacterial inhibition, particularly against *P. aeruginosa*. This comparison suggests that lignin-PLGA-FF NPs could be a highly effective antimicrobial formulation. The observed performance might be related to enhanced delivery and possibly increased stability or uptake by bacterial cells, as well as the intrinsic antibacterial properties of lignin, warranting further investigation into the mechanisms driving this increased efficacy. Lignin and its derivatives have been shown to possess antimicrobial properties against a variety of microorganisms (Ullah et al. 2022). The mechanism underlying the antimicrobial effect is not fully understood, but it is believed to involve disruption of microbial cell walls and inhibition of enzyme activity essential for microbial growth (Ullah et al. 2022). Studies have demonstrated the efficacy of lignin-derived products against bacteria, fungi, and yeast, suggesting their broad-spectrum antimicrobial activity (Chen et al. 2023). This property makes lignin a promising candidate for the development of new antimicrobial agents and coatings, especially in an era in which antibiotic resistance is a growing concern (Vinardell and Mitjans 2017).

The effectiveness of lignin-PLGA-FF NPs against biofilm formation by the selected bacterial strains may offer a promising approach for addressing persistent infections that are often resistant to conventional treatments. The enhanced inhibition of biofilm formation by lignin-PLGA-FF nanoparticles, compared to PLGA-FF nanoparticles and free FF, indicates that the encapsulation of FF also potentially enhances its bioavailability and efficacy against biofilms. This difference in effectiveness can be attributed to the higher content of FF in the lignin-PLGA-FF nanoparticles, which is significantly higher than that in the PLGA-FF nanoparticles because of the higher loading capacity, despite the same starting nanoparticle concentration.

The findings related to the effect on the preformed biofilm corroborated previous research (Yuan et al. 2020; Trif et al. 2023a, b) suggesting that FF, regardless of the delivery form, is ineffective against biofilms that have been allowed to form over a 24-hour period, even at high concentrations.

### Conclusion

This study successfully demonstrated the synergistic potential of combining PLGA with lignin and forming nanoparticles to enhance the pharmacological profile of nanodelivered FF in veterinary applications. This innovative approach not only capitalizes on the biocompatible and biodegradable nature of PLGA, but also leverages the properties of lignin, presenting a composite material that excels in controlled drug release, stability, and efficiency. The findings revealed that lignin-PLGA-FF NPs significantly improved the delivery and efficacy of FF, facilitating a reduction in the necessary dosages, thereby mitigating the risk of antimicrobial resistance development. Furthermore, the ability of the lignin-PLGA system to offer controlled release extended the utility of FF, ensuring a sustained therapeutic effect. Notably, lignin-PLGA nanoparticles demonstrated minimal cytotoxic effects, reaffirming the safety profile of thesenanoparticles for veterinary use. The lowered MIC of the nanodelivered FF when compared to its free form signifies a marked improvement in antimicrobial performance. Additionally, the capacity of this system to partially inhibit bacterial biofilm formation indicates its effectiveness in preventing the establishment of bacterial colonies that are resistant to conventional treatments. Although this study has laid the groundwork for a promising alternative in the fight against bacterial pathogens across multiple animal species, it also opens avenues for future research. Investigating the long-term effects of the lignin-PLGA nanoparticle system, exploring its applicability across a broader spectrum of antibiotics, and assessing its performance in real-world veterinary settings are essential. In conclusion, the lignin-PLGA-based loading system for FF represents a significant advancement in veterinary medicine, offering a viable solution for the pressing issues of drug efficacy, safety, and the ever-looming threat of AMR. By pushing the boundaries of nanotechnology, there is a pathway to more effective, efficient, and sustainable antibiotic treatments, marking a stride towards safeguarding the health and well-being of animals worldwide.

## Data Availability

No datasets were generated or analysed during the current study.

## References

[CR1] Adamo G, Campora S, Ghersi G (2017) Chap. 3 - Functionalization of nanoparticles in specific targeting and mechanism release. In Ficai D, Grumezescu M (ed) Nanostructures for novel therapy. 10.1016/B978-0-323-46142-9/00003-7

[CR2] Albanese A, Tang PS, Chan WCW (2012) The effect of nanoparticle size, shape, and surface chemistry on biological systems. Annu Rev Biomed Eng 14:1–16. 10.1146/annurev-bioeng-071811-150124 22524388 10.1146/annurev-bioeng-071811-150124

[CR3] Alshawwa SZ, Kassem AA, Farid RM, Mostafa SK, Labib GS (2022) Nanocarrier drug delivery systems: characterization, limitations, future perspectives and implementation of artificial intelligence. Pharmaceutics 14(4):883. 10.3390/pharmaceutics14040883 10.3390/pharmaceutics14040883 PMC9026217 35456717

[CR4] Arriagada F, Günther G, Zabala I, Rubio-Retama J, Morales J (2019) Development and characterization of Florfenicol-loaded BSA nanoparticles as controlled release carrier. AAPS PharmSciTech 20:202 10.1208/s12249-019-1419-7 31140015

[CR5] Astete CE, De Mel JU, Gupta S, Noh YR, Bleuel M, Schneider GJ, Sabliov CM (2020) Lignin-Graft-Poly(lactic- co-glycolic) acid biopolymers for polymeric nanoparticle synthesis. ACS Omega 5(17):9892–9902. 10.1021/acsomega.0c00168 10.1021/acsomega.0c00168 PMC7203963 32391476

[CR6] Bardhan A, Abraham TJ, Singha J, Rajisha R, Krishna EKN, Panda SK, Patil PK (2023) Impacts of oral florfenicol medication and residues on the kidney and liver of Nile Tilapia Oreochromis Niloticus (L). Vet Sci 10(1):36. 10.3390/vetsci10010036 10.3390/vetsci10010036 PMC9863828 36669037

[CR7] Beck-Broichsitter M, Ruppert C, Schmehl T, Guenther A, Betz T, Bakowsky U, Seeger W, Kissel T, Gessler T (2011) Biophysical investigation of pulmonary surfactant surface properties upon contact with polymeric nanoparticles in vitro. Nanomedicine 7(3):341–350. 10.1016/j.nano.2010.10.007 21059405 10.1016/j.nano.2010.10.007

[CR8] Cerbu C, Kah M, White JC, Astete CE, Sabliov CM (2021) Fate of biodegradable engineered nanoparticles used in veterinary medicine as delivery systems from a one health perspective. Molecules 26(3). 10.3390/molecules26030523 10.3390/molecules26030523 PMC7863917 33498295

[CR9] Champion JA, Mitragotri S (2006) Role of target geometry in phagocytosis. Proc Nat Acad Sci U S A 103(13):4930–4934. 10.1073/pnas.0600997103 10.1073/pnas.0600997103 PMC1458772 16549762

[CR10] Chen M, Li Y, Liu H, Zhang D, Shi QS, Zhong XQ, Guo Y, Xie XB (2023) High value valorization of lignin as environmental benign antimicrobial. Mater Today Bio 18:100520. 10.1016/j.mtbio.2022.100520 36590981 10.1016/j.mtbio.2022.100520 PMC9800644

[CR11] Chithrani BD, Ghazani AA, Chan WCW (2006) Determining the size and shape dependence of gold nanoparticle uptake into mammalian cells. Nano Lett 6(4):662–668. 10.1021/nl052396o 16608261 10.1021/nl052396o

[CR12] Costa GA, Rossatto FCP, Medeiros AW, Correa APF, Brandelli A, Frazzon APG, Da Motta AS (2018) Evaluation antibacterial and antibiofilm activity of the antimicrobial peptide P34 against Staphylococcus aureus and Enterococcus faecalis. Acad Bras Cienc 90(1):73–84. 10.1590/0001-3765201820160131 10.1590/0001-3765201820160131 29424388

[CR13] Danhier F, Ansorena E, Silva JM, Coco R, Le Breton A, Préat V (2012) PLGA-based nanoparticles: an overview of biomedical applications. J Control Release 161(2):505–522. 10.1016/j.jconrel.2012.01.043 22353619 10.1016/j.jconrel.2012.01.043

[CR14] Desai MP, Labhasetwar V, Walter E, Levy RJ, Amidon GL (1997) The mechanism of uptake of biodegradable microparticles in Caco-2 cells is size dependent. Pharm Res 14(11):1568–1573. 10.1023/A:1012126301290 9434276 10.1023/a:1012126301290

[CR15] Devadasu VR, Bhardwaj V, Kumar MNVR (2013) Can controversial nanotechnology promise drug delivery? Chem Rev 113(3):1686–1735. 10.1021/cr300047q 23276295 10.1021/cr300047q

[CR16] Dupuis V, Cerbu C, Witkowski L, Potarniche AV, Timar MC, Żychska M, Sabliov CM (2022) Nanodelivery of essential oils as efficient tools against antimicrobial resistance: a review of the type and physical-chemical properties of the delivery systems and applications. Drug Deliv 29(1):1007–1024. 10.1080/10717544.2022.2056663 35363104 10.1080/10717544.2022.2056663 PMC8979527

[CR17] González-Martín JV, Elvira L, Cerviño López M, Pérez Villalobos N, Calvo López-Guerrero E, Astiz S (2011) Reducing antibiotic use: selective metaphylaxis with florfenicol in commercial feedlots. Livest Sci 141(2–3):173–181. 10.1016/j.livsci.2011.05.016

[CR18] Gumustas M, Sengel-Turk CT, Gumustas A, Ozkan SA, Uslu B (2017) Effect of polymer-based nanoparticles on the assay of antimicrobial drug delivery systems. In Grumezescu M (ed) Multifunctional systems for combined delivery, biosensing and diagnostics 67–108. 10.1016/B978-0-323-52725-5.00005-8

[CR19] Harush-Frenkel O, Bivas-Benita M, Nassar T, Springer C, Sherman Y, Avital A, Altschuler Y, Borlak J, Benita S (2010) A safety and tolerability study of differently-charged nanoparticles for local pulmonary drug delivery. Toxicol Appl Pharmacol 246:83–90. 10.1016/j.taap.2010.04.011 20417650 10.1016/j.taap.2010.04.011

[CR20] Honary S, Zahir F (2013) Effect of zeta potential on the properties of nano-drug delivery systems - a review (part 1). Trop J Pharml Res 12(2). 10.4314/tjpr.v12i2.19

[CR21] Hoshyar N, Gray S, Han H, Bao G (2016) The effect of nanoparticle size on in vivo pharmacokinetics and cellular interaction. Nanomedicine 11(6):673–692. 10.2217/nnm.16.5 10.2217/nnm.16.5 PMC5561790 27003448

[CR22] Hu D, Cao S, Zhang G, Xiao Y, Liu S, Shang Y (2017) Florfenicol-induced mitochondrial dysfunction suppresses cell proliferation and autophagy in fibroblasts. Sci Rep 7(1). 10.1038/s41598-017-13860-9 10.1038/s41598-017-13860-9 PMC5648778 29051574

[CR23] Kang BS, Choi JS, Lee SE, Lee JK, Kim TH, Jang WS, Tunsirikongkon A, Kin JK, Park JS (2017) Enhancing the in vitro anticancer activity of albendazole incorporated into chitosan-coated PLGA nanoparticles. Carbohydr Polym 159:39–47. 10.1016/j.carbpol.2016.12.009 10.1016/j.carbpol.2016.12.009 28038752

[CR24] Karp F, Busatto C, Turino L, Luna J, Estenoz D (2019) PLGA nano- and microparticles for the controlled release of florfenicol: experimental and theoretical study. J Appl Pol Sci 136(12). 10.1002/app.47248

[CR25] Makadia HK, Siegel SJ (2011) Poly lactic-co-glycolic acid (PLGA) as biodegradable controlled drug delivery carrier. Polymers 3(3):1377–1397. 10.3390/polym3031377 10.3390/polym3031377 PMC3347861 22577513

[CR26] Mallik SK, Shahi N, Pathak R, Kala K, Patil PK, Singh B, Ravindran R, Krishna N, Pandey PK (2023) Pharmacokinetics and biosafety evaluation of a veterinary drug florfenicol in rainbow trout, Oncorhynchus mykiss (Walbaum 1792) as a model cultivable fish species in temperate water. Front Pharmacol 14. 10.3389/fphar.2023.1033170 10.3389/fphar.2023.1033170 PMC9900004 36755946

[CR27] Miyazawa T, Itaya M, Burdeos GC, Nakagawa K, Miyazawa T (2021) A critical review of the use of surfactant-coated nanoparticles in nanomedicine and food nanotechnology. *International Journal of Nanomedicine*, *16*, 3937–3999. 10.2147/IJN.S298606 10.2147/IJN.S298606 PMC8203100 34140768

[CR28] Papich MG (2016) Florfenicol. Saunders Handbook of Veterinary Drugs. Elsevier Inc. 327–329. 10.1016/b978-0-323-24485-5.00264-3

[CR29] Park BK, Lim JH, Kim MS, Hwang YH, Yun HI (2008) Pharmacokinetics of florfenicol and its metabolite, florfenicol amine, in dogs. Res Vet Sci 84(1):85–89. 10.1016/j.rvsc.2007.04.001 17570454 10.1016/j.rvsc.2007.04.001

[CR30] Patyra E, Kwiatek K (2019) HPLC-DAD analysis of florfenicol and thiamphenicol in medicated feedingstuffs. Food Addit Contam A (36)-8. 10.1080/19440049.2019.1619943 10.1080/19440049.2019.1619943 31140948

[CR31] Qi X, Jia X, Song Y (2018) Preparation and characterization of florfenicol/chitosan-stearic acid polymer nanomicelle and its antibiotic properties. J Wuhan Univ Technol Mater Sci Ed 33:1007–1013

[CR32] Raval N, Maheshwari R, Kalyane D, Youngren-Ortiz SR, Chougule MB, Tekade RK (2018) Importance of physicochemical characterization of nanoparticles in pharmaceutical product development. In: Tekade RK (ed) Basic fundamentals of drug delivery. Elsevier Inc., USA. 10.1016/B978-0-12-817909-3.00010-8

[CR33] Reda RM, Ibrahim RE, Ahmed ENG, El-Bouhy ZM (2013) Effect of oxytetracycline and florfenicol as growth promoters on the health status of cultured Oreochromis niloticus. Egypt J Aquat Res 39(4):241–248. 10.1016/j.ejar.2013.12.001

[CR34] Sandor K, Perak Junakovic E, Andrisic M, Zarkovic I, Benic M, Mihaljevic Z, Terzic S (2023) Analysis of florfenicol in pig plasma using a validated PPT-HPLC-DAD method. Veterinarska Stanica 54(3). 10.46419/vs.54.3.8

[CR35] Shrestha B, Wang L, Zhang H, Yu Hung C, Tang L (2020) Gold nanoparticles mediated drug-gene combinational therapy for breast cancer treatment. Int J Nanomed 15:8109–8119. 10.2147/IJN.S258625 10.2147/IJN.S258625 PMC7585780 33116521

[CR36] Shuang G, Yu S, Weixiao G, Dacheng W (2011) Immunosuppressive activity *of Florfenicol on the Immune responses in mice*. Immunol Invest 40(4):356–366. 10.3109/08820139.2010.551434 21314266 10.3109/08820139.2010.551434

[CR37] Sousa De Almeida M, Susnik E, Drasler B, Taladriz-Blanco P, Petri-Fink A, Rothen-Rutishauser B (2021) Understanding nanoparticle endocytosis to improve targeting strategies in nanomedicine. Chem Soc Rev 50(9):5397–5434. 10.1039/d0cs01127d 33666625 10.1039/d0cs01127d PMC8111542

[CR38] Teixeira S, Delerue-Matos C, Alves A, Santos L (2008) Fast screening procedure for antibiotics in wastewaters by direct HPLC-DAD analysis. J Sep Sci 31:2924–2931. 10.1002/jssc.200800229 10.1002/jssc.200800229 18785143

[CR39] Trif E, Cerbu C, Astete CE, Libi S, Pall E, Tripon S, Olah D, Potârniche AV, Witkowski L, Brudască GF, Spînu M, Sabliov CM (2023a) Delivery of florfenicol in veterinary medicine through a PLGA-based nanodelivery system: improving its performance and overcoming some of its limitations. Vet Res Commun. 10.1007/s11259-023-10205-y 10.1007/s11259-023-10205-y 37648880

[CR40] Trif E, Cerbu C, Olah D, Zăblău SD, Spînu M, Potârniche AV, Pall E, Brudașcă F (2023b) Old antibiotics can learn new ways: a systematic review of florfenicol use in veterinary medicine and future perspectives using nanotechnology. Animals 13(10). 10.3390/ani13101695 10.3390/ani13101695 PMC10215592 37238125

[CR41] Ullah I, Chen Z, Xie Y, Khan SS, Singh S, Yu C, Cheng G (2022) Recent advances in biological activities of lignin and emerging biomedical applications: a short review. Int J Biol Macromol 208:819–832. 10.1016/j.ijbiomac.2022.03.182 35364209 10.1016/j.ijbiomac.2022.03.182

[CR42] Uraki Y, Koda K (2015) Lignin. In: Kobayashi S, Mullen K (eds) Encyclopedia of polymeric nanomaterials. Springer, Berlin Heidelberg, pp 1073–1080. 10.1007/978-3-642-29648-2_325

[CR43] van Meerloo J, Kaspers GJL, Cloos J (2011) Cell sensitivity assays: the MTT assay. Methods Mol Biol 237–245. 10.1007/978-1-61779-080-5_20 10.1007/978-1-61779-080-5_20 21516412

[CR44] Varma KJ, Adams PE, Powers TE, Powers JD, Lamendolat JF (1986) Pharmacokinetics of florfenicol in veal calves. J Vet Pharmacol Th 9(4):412–425. 10.1111/j.1365-2885.1986.tb00062.x 10.1111/j.1365-2885.1986.tb00062.x 3806782

[CR45] Vinardell M, Mitjans M (2017) Lignins and their derivatives with beneficial effects on human health. Int J Mol Sci 18(6):1219. 10.3390/ijms18061219 28590454 10.3390/ijms18061219 PMC5486042

[CR46] Vinken M, Blaauboer BJ (2017) In vitro testing of basal cytotoxicity: establishment of an adverse outcome pathway from chemical insult to cell death. Toxicol Vitro 39:104–110. 10.1016/j.tiv.2016.12.004 10.1016/j.tiv.2016.12.004 PMC5608076 27939612

[CR47] Wei CF, Shien JH, Chang SK, Chou CC (2016) Florfenicol as a modulator enhancing antimicrobial activity: example using combination with thiamphenicol against Pasteurella multocida. Front Microbiol 7:389. 10.3389/fmicb.2016.00389 27065961 10.3389/fmicb.2016.00389 PMC4811925

[CR48] Yuan X, Liu J, Li R, Zhou J, Wei J, Jiao S, Wang ZA, Du Y (2020) Chitosan oligosaccharides coupling inhibits bacterial biofilm-related antibiotic resistance against florfenicol. Molecules 25(24). 10.3390/MOLECULES25246043 10.3390/molecules25246043 PMC7767115 33371321

[CR49] Zhang Q, Tang SS, Qian MY, Wei L, Zhou D, Zhang ZJ, He JK, Zhang QJ, Zhu P, Xiao XL (2016) Nanoemulsion formulation of florfenicol improves bioavailability in pigs. J Vet Pharmacol Ther 39:84–89. 10.1111/jvp.12230 10.1111/jvp.12230 25891823

